# Gradient to sectioning CUBE workflow for the generation and imaging of organoids with localized differentiation

**DOI:** 10.1038/s42003-023-04694-5

**Published:** 2023-03-21

**Authors:** Isabel Koh, Masaya Hagiwara

**Affiliations:** Cluster for Pioneering Research, RIKEN, Saitama, 351-0198 Japan

**Keywords:** Tissue engineering, Lab-on-a-chip, Differentiation

## Abstract

Advancements in organoid culture have led to various in vitro mini-organs that mimic native tissues in many ways. Yet, the bottleneck remains to generate complex organoids with body axis patterning, as well as keeping the orientation of organoids during post-experiment analysis processes. Here, we present a workflow for culturing organoids with morphogen gradient using a CUBE culture device, followed by sectioning samples with the CUBE to retain information on gradient direction. We show that hiPSC spheroids cultured with two separated differentiation media on opposing ends of the CUBE resulted in localized expressions of the respective differentiation markers, in contrast to homogeneous distribution of markers in controls. We also describe the processes for cryo and paraffin sectioning of spheroids in CUBE to retain gradient orientation information. This workflow from gradient culture to sectioning with CUBE can provide researchers with a convenient tool to generate increasingly complex organoids and study their developmental processes in vitro.

## Abstract (web summary)

Using a CUBE culture device, the workflow for the culture of organoids permits retention of gradient direction and differentiation marker expression to follow complex developmental progress in vitro.

## Introduction

Pluripotent stem cells (PSCs) and organoids derived from them offer a pragmatic way to model and study the formation of tissues and organs during early human development, as real specimens pose challenging ethical issues^1–4^. Protocols to generate the various organoids that mimic native tissues generally rely on sequential manipulation of the activation or inhibition of signalling pathways such as Nodal, Hedgehog, Notch, Wnt, or BMP at different time points to induce differentiation towards specific lineages, such as in the generation of intestine^5^, kidney^6^, and lung^7^ organoids, epiblasts^8,9^, and gastruloids^10^.

Nevertheless, controlling the growth and differentiation of cells along a body axis remains a challenge in 3D organoid cultures. In vitro, anterior–posterior and dorsal–ventral patterning in organoids can arise by cellular self-organization^11,12^, or achieved by fusing separately differentiated organoids together as an assembloid such as in cerebral organoids with different brain regions or biliary organoids with liver, biliary tract and pancreas components^13,14^. The limitation with these methods, though, is that because the cells are cultured in a single uniform medium, the level of control in terms of spatial information supplied to the cells is quite low; all cells within the cluster of cells that make up the organoid receive the same differentiation cues from the signalling molecules in the medium. In vivo, on the other hand, concentration gradients of morphogens from other sources also contribute to governing where cells go and what phenotype or pattern they should adopt during development^15,16^. For example, the formation of the neural tube is reliant on signals from the notochord and non-neural ectoderm layer^17^, and the nephrons of the kidney develop by exchange of various signals between the ureteric bud and metanephric mesenchyme^18^. Thus, there is a need to replicate morphogen gradients in order to generate organoids that more closely resemble the native tissues in vivo.

Several engineering technologies have been developed to mimic supplying spatial gradient of signalling molecules to cells in vitro. PSCs engineered to express Sonic Hedgehog (Shh)^19^ or agarose beads soaked with morphogens^20^ can be placed close to the developing organoid and the diffusion of molecules from the source to the differentiating cells created a high-to-low concentration gradient of morphogens. Additionally, various modified transwells and microdevices that enable cells to be cultured with two separate media compartments have also been developed to generate morphogen gradients in opposing directions across the cells^21–26^. However, these technologies are not without drawbacks: (1) they require complicated preparation and setup procedures which are not easy to perform in most biology-based laboratories without specialist skills and equipment, (2) they lack control over the placement and positioning of the sample in the device, and (3) it is difficult to retrieve the sample from the device post-experiment for further analyses without causing a lot of damage to the sample or losing the orientation of the sample. In particular, the ability to retain information on the orientation of the gradient that cells were subject to is critical to ensure proper analysis of the sample.

**Fig. 1:**
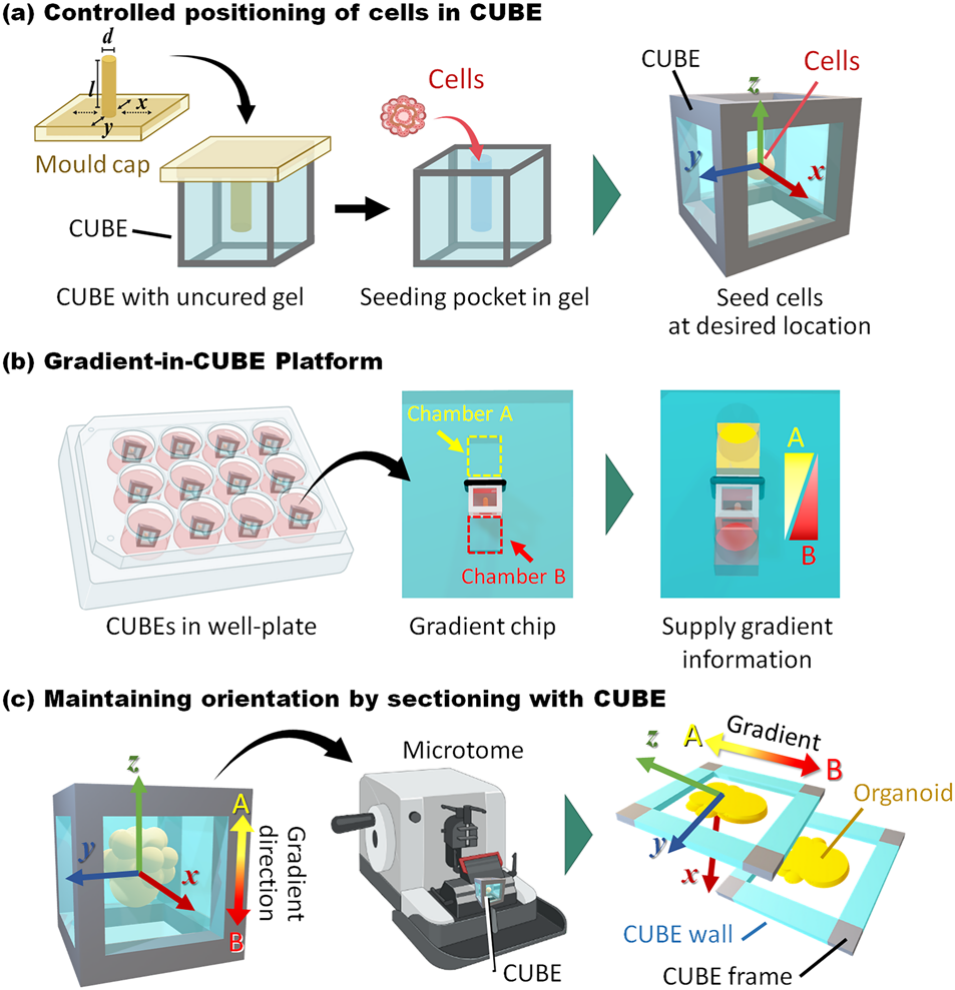
Workflow from gradient culture to sectioning for imaging. The concept for this work was to utilise the CUBE culture device to **a** firstly control the seeding position of cells at the desired location, **b** then transfer the CUBE to a Gradient-in-CUBE chip to culture cells with a morphogen gradient, and **c** finally section the sample with the CUBE to maintain gradient orientation information in the sections.

We therefore aimed to address these issues by developing a simple easy-to-use gradient culture platform to control the differentiation of PSCs into organoids with distinct localized patterns, whilst also retaining sample integrity and gradient orientation for imaging analysis processes. To achieve this, we make use of a CUBE culture device previously developed to enhance the handling ability of samples cultured in ECM hydrogel and repeatability of cell seeding and pattern formation^27,28^. Due to the simplicity of the CUBE device that comprises a simple hard material frame with see-through walls that allow visibility of the sample within, the design and composition of the CUBE can be easily tailored to fit the requirements of the experiment. For example, in this study, the frame design was modified to ensure the water-tight integrated with a fluidic device, and frame material chosen to be compatible with the organic reagents used in post-experiment sample processing. Here, we first show how we can precisely position iPSC cell spheroids in the desired position in the CUBE. Then, we demonstrate the ease of which gradient can be generated in the CUBE to induce localized differentiation of iPSCs simply by transferring it to a two-compartment gradient chip device without the need to set up complicated pump systems. Finally, we present the compatibility of the gradient platform with different post-experiment processing methods (cryo and paraffin sectioning) for imaging and analysis whilst maintaining the gradient orientation information (Fig. 1). With this Gradient-in-CUBE workflow, organoids with controlled body axis differentiation can be achieved, providing ever more complex in vitro models in which we can systematically apply and study the effects of morphogen gradients in directing cell differentiation and development into tissues, organs, and eventually body systems to further our understanding of the developmental processes in human.

## Results

Gradients of morphogen signalling contribute to cell fate specification and patterning in developing tissues^29^, and recapitulating this phenomenon in vitro could promote the development of increasingly complex organoids models. However, differentiation by gradient cues is difficult to achieve in organoids that are cultured in a single uniform medium in a well-plate and rely mainly on self-organization for patterning^30^. Although there have been many reports on methods to culture organoids with morphogen gradients^19,20,31^, the complicated setup and poor sample handling ability, as well as maintenance of gradient orientation during analysis, limit their widespread adoption by the organoid community. By taking advantage of the easy handling ability of the CUBE device, we established a workflow from culturing organoids with morphogen gradient to imaging samples with gradient orientation, with the following processes: (1) control the initial seeding position of cells in the CUBE that allow cells to receive consistent gradient information in each experiment, (2) generate a gradient of morphogen signalling along an axis of the cell sample, and (3) section the sample with the CUBE to retain information of the gradient direction.

### Precise seeding positioning in CUBE

**Fig. 2:**
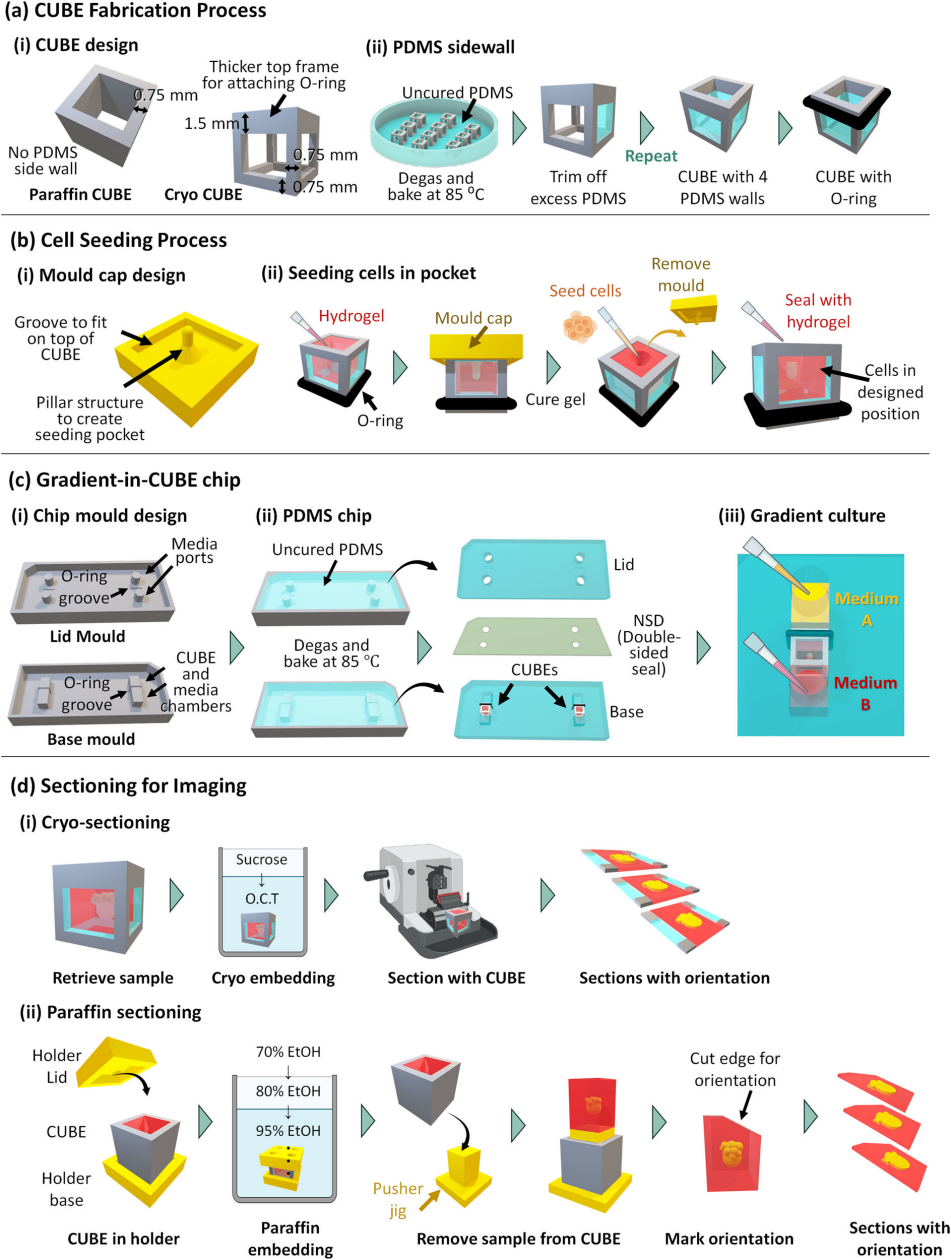
Schematic diagram of fabrication and methods processes. **a** CUBE fabrication process (i) CUBE designs for paraffin and cryo sectioning. (ii) Process to adhere PDMS sidewalls to CUBE device. **b** Cell seeding process (i) Mould cap design. (ii) Process to seed cells in the seeding pocket created by the mould cap in the hydrogel in the CUBE. **c** Gradient-in-CUBE fabrication process (i) Designs for moulds to fabricate the lid and base of the chip. (ii) Process to fabricate PDMS chips from moulds, and adhering the lid to the base by NSD, a double-sided PDMS adhesive seal. **d** Sectioning for imaging. (i) Process to embed samples in cryo medium and sectioning with CUBE. (ii) Process to embed samples in paraffin with CUBE holder, then sectioning with a marked edge to maintain orientation of sample.

The precise placement of cells in the CUBE is important to ensure consistency in the gradient signalling that cells receive. For example, the gradient information sensed by cells that were placed too close to one end of the CUBE will be different from that sensed by cells in the centre of the CUBE. To control the seeding position of cells in the CUBE, a mould cap with a pillar structure to create a seeding pocket at the desired position in the hydrogel in the CUBE, as well as grooves to ensure the precise fitting of the mould cap on the CUBE was designed (Fig. 2b(i)). The process to make a seeding pocket and seed cell spheroids in the pocket is illustrated in Fig. 2b(ii) and described in the methods section. By utilizing this seeding method, cells can always be seeded in the desired location with low variation, compared to manually positioning the cells without a guide which results in inaccurate seeding with high variation (Supplementary Fig. 1). Although it could be argued that a highly trained person or a robot may be able to position cells in the uncured gel with high accuracy without the need of the mould cap, the gelation behaviour of soft hydrogels such as collagen or Matrigel can vary a lot depending on temperature and handling of the sample, and it is difficult to predict when the gel will have polymerized sufficiently to hold the cells in position. To highlight the ease in which this method can be utilized by anyone, we recruited our lab secretary and a junior high school student, both of whom had no experience with using the CUBE or the mould cap, to perform the same experiment. With minimal training, both succeeded in seeding with accuracy on a similar level to an experienced user, and with significantly higher accuracy compared to an experienced user without the mould cap (Supplementary Fig. 1).

### Gradient generation and localized differentiation using Gradient-in-CUBE chip

To establish a gradient across the length of the CUBE, a Gradient-in-CUBE chip, comprising a base component and a lid component, was designed and fabricated by a moulding process. The mould for the base contained a compartment for the CUBE and two separate media chambers, as well as a groove to fit the O-ring; the mould for the lid has two ports for adding media to the two separate media chambers (Fig. 2c(i)). The processes to make the chip are illustrated in Fig. 2c(ii), (iii) and described in the methods section, with a Supplementary Movie 1 showing how the CUBE is integrated with the chip.

**Fig. 3:**
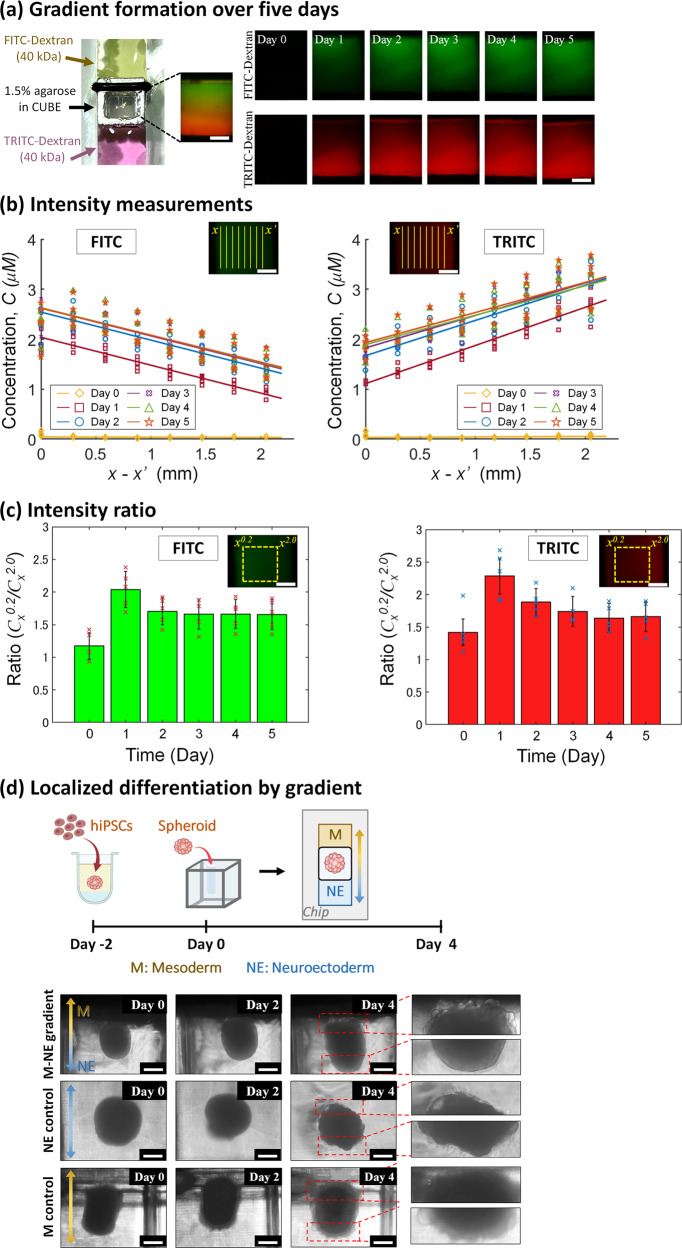
Generating gradient and localized differentiation using the Gradient-in-CUBE Chip. **a** Imaging of FITC- and TRITC-dextran gradient forming over a five-day period. Imaging was performed every 24 hours after the used dextran was removed, the media chamber washed with DPBS, and fresh dextran added to the chamber. The dimensions of the window of the CUBE were w = 2.75 mm; h = 3.5 mm and the imaging field of view at 4x magnification was *w* = 3.6 mm; *h* = 2.7 mm. Scale bar = 1 mm. **b** Concentration, *C* was determined by linearly correlating the average intensity along the y-axis for each pixel in the centre region (w = 2.2 mm; h = 2.2 mm) of the fluorescence image from *x* to *x*’ to that obtained from a standard curve fitting of FITC- and TRITC-dextran concentrations. Scale bar = 1 mm. Markers represent selected data points at every 50 pixels (~0.3 mm), and lines show the linear least squares best fit; *n* = 5. The slopes of individual lines for FITC were −0.0044 for Day 0, −0.5586 for Day 1, −0.5607 for Day 2, −0.5549 for Day 3, −0.5487 for Day 4, and −0.5391 for Day 5. For TRITC, the slopes were 0.0095 for Day 0, 0.7644 for Day 1, 0.7062 for Day 2, 0.6504 for Day 3, 0.5897 for Day 4, and 0.6039 for Day 5. Day 0 shows little gradient, but by day 1 a concentration gradient is generated from one end of the CUBE to the opposite end for FITC and TRITC, respectively, in the opposite direction. **c** The ratio between source-end concentration and sink-end concentration was taken as a representation of gradient steepness. The area analysed was from *x* = 0.2 mm to *x* = 2.0 mm as the approximate region in which the spheroid would be located. The gradient was at its steepest on day 1, but although the steepness decreased from day 2, a gradient can be consistently maintained for five days. **d** Differentiation of hiPSC spheroid with mesoderm-inducing medium (M) on one side and neuroectoderm-inducing medium (NE) on the opposite side results in a spheroid with different morphology on either end of the spheroid, whereas the morphology is relatively uniform in M or NE only controls. Scale bar = 500 μm.

FITC-dextran and TRITC-dextran were used to simulate the addition of two different types of media to the CUBE, and daily imaging was performed to monitor the progress of the dual gradient formation in the CUBE over a 5-day period (Fig. 3a). The average concentration (*C*) of FITC and TRITC across the centre region of the window of the CUBE (*x – x’*) were measured, and showed an opposing gradient of high concentration at the source side to lower concentration at the sink side, as the respective dextran molecules move from the side where it is highly concentrated, to the opposite side where the concentration is much lower (Fig. 3b). 10 μM of dextran was added to the source reservoir, and the average maximum concentration at the source-end of the FOV was FITC = 2.618 μM and TRITC = 3.255 μM, whereas the minimum at the sink-end was FITC = 0.024 μM and TRITC = 0.026 μM. The gradient was calculated as the ratio of *I*_*x*_^0.2^/*I*_*x*_^2.0^ for FITC and *I*_*x*_^2.0^/*I*_*x*_^0.2^ for TRITC, where a value of one shows no gradient and a higher ratio represents a steeper gradient (Fig. 3c). The steepness of the gradient increases and peaks over the first 24 hours as the molecules begin to enter the gel, but as the molecules accumulate in the opposite side as well as in the gel in the CUBE, the steepness of the gradient decreases by day 2. Nevertheless, with daily rinsing with DPBS and replacement with fresh dextran, a constant gradient can be sustained for 5 days without the media reaching a state of equilibrium. In this study, only the diffusion of 40 kDa dextran into an agarose gel was used to model the diffusion of growth factors into an ECM hydrogel. These were chosen on the basis that the molecular weight of Wnt, which plays an important signalling role in development, is about 40 kDa, and that dextran and agarose are readily available reagents which have been used in numerous mass transport and diffusion studies. Due to the large number of known morphogen gradients with varying molecular weights, and the vast choice of hydrogel available to use in cell culture, it was not plausible to investigate the various permutations of morphogen and hydrogel pairings in the present study.

Given that a gradient across a scale of a single or a few cells’ length is sufficient to provide positional information to cells^32,33^, we postulated that the gradient generated in the Gradient-in-CUBE chip should be sufficient to induce localized differentiation on opposite ends of a spheroid. Additionally, another strategy to counteract the accumulation of morphogen is to supplement inhibitors of the morphogen to the opposing side of the gradient, as the interplay between morphogen and its inhibitor is also critical for patterning in tissues in vivo. For example, the Wnt and Nodal antagonists Dkk1, Lefty-1, and Cer-1 in the anterior parts of the embryo restricts Wnt/Nodal to the posterior end of the embryo to establish the anterior–posterior axis^34^.

To demonstrate the application of the Gradient-in-CUBE chip to differentiate a single spheroid into two localized regions, we supplied a neuroectoderm differentiation medium (NE) to one end of the CUBE, and mesoderm differentiation medium (M) on the opposite side. The mesoderm medium (based on a protocol by Lam et al.^35^) contained high concentrations of Wnt activator CHIR99021, whereas the neuroectoderm medium (based on a protocol by Bianchi et al.^36^) contained lower Wnt activator along with Nodal/Activin inhibitor SB431542 to counteract mesoderm differentiation on the neuroectoderm side. After 4 days of differentiation, morphological differences could be observed at both ends of the spheroid, with the mesoderm side having a bumpier and more protruding feature, compared to smoother feature of the neuroectoderm side. In contrast, control spheroids showed rather uniform morphological features—mesoderm control had bumpy protrusions all over the surface; neuroectoderm control had smoother surface (Fig. 3d).

### Maintaining orientation during sectioning and imaging

It is often difficult to visualize the expression of cell pluripotency or differentiation markers in larger scale 3D samples like organoids, due to the limited range of laser penetration, as well as the low sensitivity of low magnification objective lenses and short focal lengths of high-sensitivity lenses. Hence, organoids are often embedded in cryo medium or paraffin and sliced into thin sections for staining and imaging. However, the orientation of the sample is often lost after retrieving the sample from prevailing gradient-generating devices. The advantages of the CUBE device are not only that the cells are contained within the CUBE and can be retrieved without causing damage to the sample, but also that the orientation of the gradient can easily be marked to be recognized later. Here, we show that the samples in the CUBE can be processed for cryo and paraffin sectioning.

**Fig. 4:**
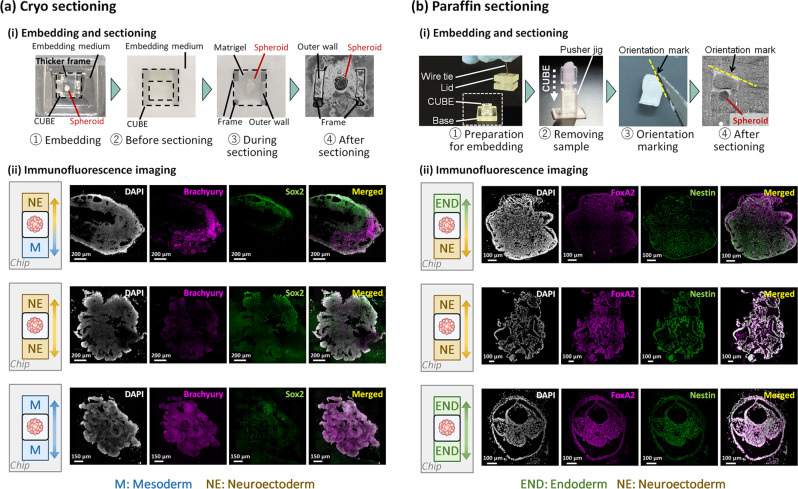
Immunofluorescence imaging after cryo and paraffin sectioning. **a** Cryo sectioning and imaging. (i) Steps to embed and section frozen samples with CUBE, making use of the CUBE frame as a reference marker for sample orientation. (ii) Immunostaining of hiPSC spheroid differentiated with M-NE gradient showed localized expression pattern of mesoderm (Brachyury) and neuroectoderm (Sox2) markers, whereas NE only and M only controls showed uniform distribution of the markers. **b** Paraffin sectioning and imaging. (i) Steps to embed and section paraffin samples with CUBE holder to prevent sample loss, then removing sample from CUBE and cutting an edge of the sample to mark the orientation. (ii) Immunostaining of hiPSC spheroid differentiated with endoderm (END)-NE gradient showed localized expression pattern of endoderm (FoxA2) and neuroectoderm (Nestin) markers, whereas NE only and END only controls showed uniform distribution of the markers.

For cryo sectioning, the thicker frame on one end of the CUBE where the O-ring was attached acts as an orientation marker. After retrieving the sample from the Gradient-in-CUBE chip, the CUBE can simply be soaked in sucrose and cryo embedding medium before freezing. Once frozen, the CUBE is sliced together with the sample, with the CUBE frame and PDMS outer wall acting as a reference to preserve sample orientation (Figs. 2d(i) and 4a). From immunofluorescence staining of hiPSC spheroid differentiated with NE-M gradient, localization of neuroectoderm marker Sox2 and mesoderm marker Brachyury were observed on the respective opposite ends of the spheroid, whereas in the control samples, both markers were expressed uniformly throughout the spheroid (Fig. 4b). Thus, we demonstrated a method to preserve the orientation information of spheroid differentiated with morphogen gradient by sectioning the sample as is in the CUBE. A downside to this method, however, is that the microtome blade gets damaged by repeated cutting of the hard acrylic material and may need to be replaced frequently. This issue may be resolved by using a softer material for the CUBE frame that is easier to cut, such as polycarbonate or Teflon. Alternatively, saw microtomes or diamond-coated blades that are commonly used for sectioning hard samples like bone could be used to cut through the CUBE frame.

For paraffin sectioning, some extra steps have to be taken to preserve the integrity and gradient orientation of the sample in the CUBE (Figs. 2d(ii) and 4b(i) and Supplementary Fig. 2). While sectioning with the CUBE could be done with the cryo CUBE which is made of a combination of acrylic frame and PDMS wall which is a soft material, it is much more difficult to section the paraffin CUBE, as the whole CUBE is made of acrylic, which was a necessary modification because PDMS is not compatible with organic solvents used in the paraffin embedding process. During the initial dehydration process prior to paraffin embedding, the hydrogel loses a lot of volume and shrinks in the CUBE. To avoid the risk of losing the sample that may detach from the CUBE, a CUBE holder comprising a lid and a base was designed to cover most of the top and bottom open surfaces of the CUBE but still allow reagents to pass through in and out of the sample. The CUBE in the holder was then tied with a wire to keep them together. After the paraffinization process, the sample was removed from the CUBE using a pusher jig, and the orientation marked by cutting an edge on one corner of the sample. To demonstrate the application of the paraffin method, hiPSC spheroid was differentiated with NE and endoderm (END) differentiation (based on a protocol by Lam et al.^35^) media on either side of the CUBE. Immunofluorescence staining of neuroectoderm markers Nestin and Sox2, and endoderm markers FoxA2 and Sox17, showed localization on the NE and END sides of the spheroids, respectively (Fig. 4b(ii) and Supplementary Fig. 3). On the other hand, control samples without gradient culture showed uniform expression of the markers throughout the spheroid.

## Discussion

The FITC/TRITC-dextran and NE/M as well as END/NE differentiation experiments here demonstrated that morphogen gradients can be generated in the CUBE, and can be used to control the differentiation of cell spheroids. It should be noted, however, that the gradient generated by the platform presented in this paper relies only on the free diffusion of molecules from the source, across the gel in the CUBE, to the sink. As the rate of free diffusion is dependent on the size, charge, and solubility of the solute^37^, as well as the pore size, charge, and polymer mobility of the hydrogel matrix^38^, detailed experimental or simulation studies taking into account the specific morphogen-generating molecule, the diffusivity of each molecule and how hydrogel properties affect the diffusion of each molecule^39–45^ may be necessary to understand the minute details of mass transport to achieve even finer control of gradient generation, but this level of precision is beyond the scope of this study. Alternatively, ensuring a constant concentration of growth factors in the medium is one way to better control the accuracy of the gradient, for example with more frequent change or mixing of the medium, or by pumping a constant flow of fresh medium to the sample, although these methods would increase the complication of the setup and culture procedures.

The role and mechanisms of morphogen gradients in guiding cell fate is complex and still not fully understood. When cells undergo self-organization to form specific patterns of the tissue, both local gradients generated by secretions of the cells themselves and their immediate surrounding neighbours, as well as external gradients in the ECM surrounding the cells generated by cells that are further away such as those in the surrounding mesenchyme, contribute to governing cell differentiation^15,16^. Theoretical models such as the reaction-diffusion, positional information (French flag), or synthesis-diffusion-clearance models have been used to explain or predict the short- and long-range interactions between cells and morphogen and the resulting pattern formation^29,45,46^, but these studies are focussed on the local environment of the cells. On the other hand, the CUBE gradient platform aims to mimic the supply of external gradients by other populations of cells in the periphery that play a role in forming the higher-order structures of organs, such as in the branching patterns of mammary gland, kidney, or lung^47–49^. Hence, detailed optimization into the interaction between cells and gradients of various morphogens, as well as the timing of gradient formation would be required to develop specific target organoids with the desired pattern. Furthermore, even though the growth factors added to both ends of the control samples were exactly the same, gradients of the growth factors would also exist as they entered the gel from the opposite ends, and longer-term studies of how this may affect the self-organization of cells within larger and smaller spheroids would be needed in the future.

As the CUBE and gradient chip design can be customized according to the user’s needs, the platform could also be applicable for developing highly complex organoids such as by applying gradients to four sides of the CUBE to generate organoids with anterior–posterior and dorsal–ventral axes.

One of the major drawbacks of using PDMS to fabricate the gradient chip is the absorption of proteins and small molecules to the PDMS surface due to its hydrophobic nature^50–52^. Despite this, PDMS has its many advantages such as biocompatibility, gas permeability, optical transparency, and easy fabrication process. There are also several relatively simple methods that have been reported to reduce protein absorption, including mixing PDMS with poly(ethylene glycol) (PEG) to increase the hydrophilicity of PDMS^53^ or coating surfaces with Teflon, 2-methacryloyloxyethyl phosphorylcholine (MPC) polymer or paraffin wax^54–56^.

Although immunofluorescence staining of the differentiation markers show localised differentiation of the spheroid, the structure and morphology of each sample are varied, such as in the case of endoderm control in Fig. 4b(ii). A possible reason for this variation is that although the positioning of cell spheroid is controlled, the initial spheroid is formed in a 96-well plate with no control of spheroid shape. With several studies reporting the contribution of geometric constrains in mimicking patterning and symmetry breaking events of development in in vitro culture^57,58^, it may be better to apply the mould cap method to position single cells in 3D hydrogel prior to spheroid formation in the future to control initial seeding shape such as by 3D-printing the desired geometric shape with carbohydrate glass and dissolving the carbohydrate after the gel has cured to leave behind a seeding pocket with the corresponding desired shape^59^.

An advantage of the modularity of the CUBE and chip design is that the material choice can be selected according to the needs of the experiments. For example, if optical transparency is of high priority, a CUBE with a PDMS window is more suitable; whereas if paraffinization of sample for post-experiment analysis is required, a CUBE made entirely of acrylic with no PDMS is more suitable, even though optical clarity is slightly reduced, due to the incompatibility of PDMS with organic solvents. Accordingly, the choice of material for the gradient chip does not have to be the same as that of the CUBE, and should be chosen taking into consideration the pros and cons of the material^60,61^.

Culturing 3D organoids with morphogen gradient has long been a challenge both in terms of complicated gradient device setup and maintaining the orientation of the gradient during analysis processes. In this paper, we present a gradient culture-to-imaging workflow that utilizes the modular CUBE culture device to easily set up a Gradient-in-CUBE device. Compared to gradients generated in standard microfluidic chips that can be dynamically controlled using flow and pressure, the gradient generated using our simple gradient device is less controllable over time as it relies only on the free diffusion of molecules. However, the platform presented here focusses on the usability for biologists and requires no complicated setup involving pumps or syringes. Additionally, just as the previous iteration of the CUBE has been made commercially available, the commercialization of the CUBE with various modifications to fit the users’ requirements would further increase the usability and adoption of the CUBE platform by less engineering-inclined laboratories. Furthermore, post-experiment, the sample can be easily removed from the device without damaging the sample whilst retaining information on the direction in which the gradient was formed. Although additional target organoid-dependent optimization of culture protocols may be necessary to determine the most suitable gradient slopes for specific growth factors in the chosen supporting ECM material, the straightforward and customizable methodologies in this paper have the potential to significantly advance organoid development via symmetry breaking.

An interesting point to consider in future works, however, is the potential contribution of differences in mechanical stimulation that cells experience depending on the size of the spheroids in relation to the size of the seeding pocket created by the mould cap, as the sealing of the pocket with additional hydrogel may introduce some variation in gel stiffness due to excess media in the pocket when spheroids are seeded in it. The Gradient-in-Cube platform in its current form only generates gradient in one direction, but it also has the potential to be adapted to generate gradients in two axes, for example, the anterior–posterior and dorsal–ventral axes in the same organoid, which is work that is currently being undertaken. Hence, the workflow from generation of organoid with localized differentiation to analysis with gradient information could provide researchers a user-friendly method to develop increasingly complex organoids to expand our understanding of various mechanisms such as symmetry breaking that are involved in developmental processes.

## Methods

### hiPSC maintenance and spheroid formation

Human iPSCs (IMR90-4; WiCell Research Institute; WB65317) were maintained with mTeSR Plus (STEMCELL Technologies, 100-0276) on dishes coated with 9–10 μg/cm^2^ Growth Factor Reduced Matrigel (Corning, 356231), routinely passaged using ReLeSR (STEMCELL Technologies, 05872), and cultured in a 37 °C, 5% CO_2_ incubator. Cells were tested for mycoplasma contamination using MycoAlert kit (Lonza, LT07-118). To prepare 96-well plates for spheroid formation, wells were filled with 80 μL/well of 3% agarose (Sigma, A9414) and allowed to solidify. StemFit AK02N (Ajinomoto, RC AK02N) medium plus 10 μM Rock inhibitor (Y-27632; Nacalai Tesque, 08945-84) was then added to the well and incubated. For spheroid formation, 70% confluent cells were dissociated using ReLeSR and resuspended in StemFit +Y27632 medium after centrifugation, before seeding in agarose well-plate. Cells from one 35 mm dish were used to make 5 spheroids (approximately 2–3 × 10^4^ cells per spheroid). The next day, medium was switched to mTeSR Plus medium without Y27632. Spheroids were transferred to the CUBE device after one day of culture in mTeSR Plus.

### CUBE fabrication

Material choice for the CUBE had to be carefully considered to accommodate the requirements of each procedure, particularly as the paraffinization process entails the use of organic reagents. The CUBE frame was switched from the previously used polycarbonate to acrylic material, which is more resistant to xylene treatment for short periods of time compared to polycarbonate. Additionally, polydimethylsiloxane (PDMS), a clear silicone-based biocompatible material was used as the sidewall material to restrict movement of morphogen-containing media through the CUBE to only the top and bottom sides of the CUBE, thereby generating a gradient in only one axis of the CUBE.

Two types of acrylic (poly (methyl methacrylate); PMMA) CUBEs were designed for this study using Rhinoceros 3D software (Robert McNeel & Associates). For cryo sectioning, CUBEs were designed with a thicker frame on the top side of the cube so that a nitrile O-ring (AS ONE, 62-3049-63) can be attached to the cube (Fig. 2a(i)) for water-tight sealing to reduce leakage of media around the cube during gradient culture. To make the sidewalls of the Cryo CUBE, PDMS (Silpot 184, Dow Toray, 04133124) was first prepared by mixing elastomer base with curing reagent at a 10:1 ratio. Then, a thin layer of the mixture was spread out in a petri dish and degassed to remove air bubbles. The cube frames were placed on the PDMS, then degassed again and baked at 85°C for 30 min to cure the PDMS. Once cured, the PDMS was trimmed from the frames with a scalpel, and the process was repeated to cover the other three sides of the cube, leaving the top and bottom surfaces open (Fig. 2a(ii)). The thickness of the PDMS wall is equivalent to the thickness of the CUBE frame which is 0.75 mm when ~2.8 g of PDMS per 100 mm dish was used. Because PDMS swells when exposed to xylene, CUBEs for paraffin sectioning were designed without sidewalls (Fig. 2a(i)). Although this slightly reduces the clarity of samples, a fully acrylic frame still afforded sufficient visibility under brightfield. For paraffin sectioning, CUBEs were designed as a solid cube of 5 mm length with a hollow core of 3.5 × 3.5 mm, with the thickness of the frame being 0.75 mm (Fig. 2a(i)). CUBEs for paraffin sectioning had no PDMS sidewalls as PDMS swells in xylene during the paraffinization process. All CUBEs were ordered from machining companies (Cryo CUBEs from Yumoto Electric Inc, Japan, and Paraffin CUBEs from Proto Labs, Japan). Before use, CUBEs were washed twice with ultrasonication, once with MilliQ water and once with isopropanol (IPA), then dried in the oven for 2 h.

### hiPSC seeding in CUBE

To control the positioning of cells during the initial seeding in the CUBE, a mould cap was designed with a pillar structure at the desired seeding position, and with grooves to ensure the mould fits on top of the cube so that the pillar position can be aligned properly in the cube (Fig. 2b(i)). The mould caps were printed using a 3D printer (Agilista 3200, Keyence), and cleaned by ultrasonication twice with IPA after removing excess printing support material, then dried in an oven at 65°C. Before use, the mould cap was dipped in 2-methacrylooyloxtethyl phosphorylcholine (MPC; Lipidure, NOF Corporation, CM5206E) diluted to 5% in isopropanol, then allowed to dry for 1 h at room temperature (RT). MPC coating helps to ensure smoother detachment of the mould from hydrogel. A nitrile O-ring was attached to the thick part of the acrylic frame for the Cryo CUBE, or approximately 1 mm near one end of the Paraffin CUBE. The CUBEs were placed O-ring side down in a dish, and Matrigel added into the CUBE before placing the mould cap on top of the CUBE and curing the gel in the incubator for 25 min. Once the gel has cured, the mould cap was removed, and hiPSC spheroids were seeded in the pocket created by the mould. Next, additional Matrigel was added to the top of the CUBE and allowed to cure for another 25 min to seal the pocket (Fig. 2b(ii)). After curing, the CUBE was transferred to a 48-well plate with mTeSR Plus medium and incubated for 2 h prior to starting gradient culture.

### hiPSC differentiation with Gradient-in-Chip

Moulds for making lid of PDMS gradient chips were designed with grooves to fit the O-ring and ports for adding media, while the base was designed to fit the cube, O-ring, and two separate media chambers (Fig. 2c(i)). PDMS moulds were ordered from Proto Labs. To make the PDMS chips, uncured PDMS was poured into the moulds, then degassed and baked at 85 °C for 1 h to cure the PDMS. Once cured, the chips were removed from the moulds, then washed and sterilized the same way as the CUBEs. To assemble the gradient chip, CUBEs were placed in the chip base with the O-ring fitted into the O-ring groove, and a PDMS double-sided adhesive film (NSD-100, NIPPA) with holes to access the media chambers cut into it was used to seal the lid and base together (Fig. 2c(ii)). After assembly, the media chambers on each side of the CUBE were filled with the respective differentiation media (Fig. 2c(iii)).

Neuroectoderm differentiation medium comprised 1:1 mixture of KnockOut DMEM/F12 (Gibco, 12660-012) and Neurobasal medium (Gibco, 21103-049) supplemented with 10% KnockOut serum replacement (Gibco, 10828010), 1% MEM non-essential amino acid (Nacalai Tesque, 06344-56), 1% GlutaMAX (Gibco, 35050-061), 1 μM LDN1913189 (Sigma, SML0559), 2 μM SB431542 (Nacalai Tesque, 18176-54), 3 μM CHIR99021 (Nacalai Tesque, 18764-44), 0.1 mM 2-mercaptoethanol (Nacalai Tesque, 21438-82), and 0.5 μM ascorbic acid (Nacalai Tesque, 03420-52). Mesendoderm basal medium comprised RPMI medium 1640 (Gibco, 11875-093), 1% GlutaMAX, and 1% penicillin-streptomycin (Gibco, 15140122). For mesoderm differentiation, 5 μM CHIR99021 was added to mesendoderm basal medium on Days 0 and 1. From Days 2 to 4, CHIR99021 was withdrawn and replaced with 100 ng/mL bFGF (Nacalai Tesque, 19155-36) and 1 μM all-trans retinoic acid (Stemgent, 04-0021). For endoderm differentiation, 5 μM CHIR99021 was added to mesendoderm basal medium on Day 0, and from Days 2 to 5 CHIR99021 was withdrawn and replaced with 100 ng/mL Activin A (R&D Systems, 338-AC-050/CF). Media change was performed every day by discarding spent media, washing once with DPBS, and replacing with fresh media. Phase contrast images of the spheroids in the CUBE in the chip device were taken using Olympus CKX41 microscope to monitor morphological changes to the spheroid.

### Fixation and sectioning for imaging

At the appropriate timepoint for analysis, the samples were removed from the gradient chip and transferred to a 48-well plate for fixation. Samples were washed twice with DPBS for 5 min each time, fixed with 4% paraformaldehyde for 20 min, then washed twice with DPBS for 10 min each time. For cryo sectioning, samples were soaked in 10%, 15%, and 20% sucrose (Wako, 194-00011) in succession for 1 h each, and in cryosection embedding medium (Tissue-Tek O.C.T compound; Sakura Finetek, 4583) for 10 min before embedding in embedding medium and freezing at −80 °C for 30 min. After freezing, samples were removed from the mould and sectioned at 30 μm thickness as is with the CUBE (Fig. 2d(i)) using a microtome (Yamato Kohki Industrial, Retoratome REM-710) equipped with a freezing unit (Yamato Kohki Industrial, Electro Freeze MC-802A). Sections collected on glass slides were dried with cool air for 30 min, then in an oven at 40 °C for another 30 min, followed by washing in MilliQ water to remove excess embedding medium. Excess water was removed, and the sections dried at RT for 10 min.

For paraffin sectioning, samples were dehydrated and embedded in paraffin with the following procedure: 70% ethanol for 2 h, 70% ethanol overnight, 80% ethanol for 1 h, 95% ethanol for 1 h, 99% ethanol for 1 h, 100% isopropanol for 1 h twice, 100% xylene for 1 h three times, 1:1 mixture of xylene and paraffin (Nacalai Tesque, 26029-05) overnight at 40 °C, and paraffin for 1.5 h three times at 65 °C. To prevent the loss of sample due to shrinkage of Matrigel during the dehydration process, a CUBE holder with lid and base held together by wire was designed to contain the sample in the CUBE (Fig. 2d(ii)). After the final paraffin step, the sample was removed from the CUBE holder and embedded in fresh paraffin. The CUBE was cut out from the paraffin, and the sample was removed from the CUBE by cutting the paraffin along the inner frame of the CUBE using a scalpel and pressing the CUBE onto a pusher jig (Fig. 2d(ii)). One edge of the paraffinized sample was cut to mark the orientation of gradient direction, and the sample sectioned into 10 μm thick slices using a microtome. Deparaffinization was performed as follows: 100% xylene for 10 min twice, 99% ethanol for 5 min twice, 95% ethanol for 5 min, 80% ethanol for 5 min, 70% ethanol for 5 min, MilliQ water for 5 min. Heat-induced antigen retrieval was performed by placing the samples in 10 mM citrate buffer (1.8 mM citric acid, 8.2 mM trisodium citrate, pH 6) and heating the buffer until it boils, then cooling it down for 1 min. The boiling and cooling process was repeated 6 times with 10 s of boiling followed by 1 min cool down. After the last cycle, samples were cooled for 30 min, then washed with MilliQ water for 5 min, followed by DPBS for 5 min three times.

### Immunofluorescence staining

Cryo-sectioned samples were permeabilized with 0.5% Triton X-100 for 10 min, followed by three washes with 100 mM Glycine for 10 min each time. Immunofluorescence buffer (IF buffer) was made up of 0.5% Tween20, 2% Triton X-100, and 10% bovine serum albumin (BSA; Sigma, 126615) in DPBS. Blocking was performed by incubating samples with IF buffer with 10% goat serum (Gibco, 16210064) (IF + G) for 30 min, then IF + G with 1% goat anti-mouse IgG (Bethyl Laboratories, A90-116A) for 20 min. Antibodies were diluted (1:200 or 1 μg/mL) according to Supplementary Table 1. Primary antibodies were incubated for 90 min and secondary antibodies (Alexa fluor, 1:200, Thermo Fisher Scientific)) incubated for 50 min. After each antibody incubation, samples were washed with IF buffer for 15 min three times. Nuclei were stained with DAPI for 20 min, then washed with DPBS for 5 min three times.

### FITC-dextran and TRITC-dextran gradient formation

To track the formation of gradient over a period of time, CUBE filled with 1.5% agarose was placed in the gradient chip with 10 μM 40 kDa fluorescein isothiocyanate (FITC)-dextran (Sigma, FD40S) in DPBS at one end of the CUBE, and 10 μM 40 kDa tetramethyl rhodamine B isothiocyanate (TRITC)-dextran (TdB Labs, TD40) in DPBS at the other end. Every 24 h, both FITC-dextran and TRITC-dextran were discarded, and the media chambers washed once with DPBS before replacing with fresh dextran. Following these steps, imaging at the window of the CUBE (*w* = 2.75 mm; *h* = 3.5 mm) was performed using a fluorescence microscope (BZX-700, Keyence) at ×4 magnification (field of view: *w* = 3.6 mm; *h* = 2.7 mm). The window area of the CUBE was 1.5 mm away from the source on the FITC side and 0.75 mm from the source on the TRITC side in *x*; 0.75 mm away from the CUBE wall in *y*; and 2.5 mm from the bottom of the CUBE in *z*. FITC and TRITC intensities were measured from fluorescence images using the Plot Profile tool in ImageJ software over an area in the centre of the image (*w* = 2.2 mm; *h* = 2.2 mm), which gives the average intensity along the vertical *y*-axis for each pixel in the *x*-axis. Concentration (*C*) was then calculated by liner correlation to a standard curve of FITC- and TRITC-dextran intensities of various concentrations (Supplementary Data 1). The gradient was calculated as the ratio of the concentration at 0.2 mm and at 2.0 mm (*C*_*x*_^0.2^/*C*_*x*_^2.0^ for FITC and *C*_*x*_^2.0^/*C*_*x*_^0.2^ for TRITC) to exclude the regions of at both ends of the CUBE window as the proximity to the CUBE frame affected the intensity of the dextran, and to represent the approximate region of the spheroid.

### Statistics and reproducibility

All sample sizes are larger than five and specified in figure legends. *p* value was calculated by Kolmogorov–Smirnov (KS) test in Matlab.

### Reporting summary

Further information on research design is available in the Nature Portfolio Reporting Summary linked to this article.

## Supplementary information

Supplementary Figures

Description of Additional Supplementary Files

Supplementary Data 1

Supplementary Movie 1

Reporting Summary

## Data Availability

The data that support the findings of this study are available from the corresponding author upon reasonable request. Source data underlying Fig. 3c and Supplementary Fig. 1c are provided in Supplementary Data 1.

## References

[1] Zhu Z, Huangfu D (2013). Human pluripotent stem cells: an emerging model in developmental biology. Development.

[2] van den Brink SC, van Oudenaarden A (2021). 3D gastruloids: a novel frontier in stem cell-based in vitro modeling of mammalian gastrulation. Trends Cell Biol..

[3] Huch M, Koo BK (2015). Modeling mouse and human development using organoid cultures. Development.

[4] Kim J, Koo BK, Knoblich JA (2020). Human organoids: model systems for human biology and medicine. Nat. Rev. Mol. Cell Biol..

[5] McCracken KW, Howell JC, Wells JM, Spence JR (2011). Generating human intestinal tissue from pluripotent stem cells in vitro. Nat. Protoc..

[6] Taguchi A, Nishinakamura R (2017). Higher-order kidney organogenesis from pluripotent stem cells. Cell Stem Cell.

[7] Miller AJ (2019). Generation of lung organoids from human pluripotent stem cells in vitro. Nat. Protoc..

[8] Rust WL, Sadasivam A, Dunn NR (2006). Three-dimensional extracellular matrix stimulates gastrulation-like events in human embryoid bodies. Stem Cells Dev..

[9] Simunovic M (2019). A 3D model of a human epiblast reveals BMP4-driven symmetry breaking. Nat. Cell Biol..

[10] Veenvliet, J. v. et al. Mouse embryonic stem cells self-organize into trunk-like structures with neural tube and somites. *Science* **370**, eaba4937 (2020). 10.1126/science.aba4937 33303587

[11] Moris N (2020). An in vitro model of early anteroposterior organization during human development. Nature.

[12] Zheng Y (2019). Dorsal-ventral patterned neural cyst from human pluripotent stem cells in a neurogenic niche. Sci. Adv..

[13] Bagley JA, Reumann D, Bian S, Lévi-Strauss J, Knoblich JA (2017). Fused cerebral organoids model interactions between brain regions. Nat. Methods.

[14] Koike H (2019). Modelling human hepato-biliary-pancreatic organogenesis from the foregut–midgut boundary. Nature.

[15] Christian JL (2012). Morphogen gradients in development: from form to function. Wiley Interdiscip. Rev. Dev. Biol..

[16] Solnica-Krezel L, Sepich DS (2012). Gastrulation: making and shaping germ layers. Annu. Rev. Cell Dev. Biol..

[17] Briscoe J, Small S (2015). Morphogen rules: design principles of gradient-mediated embryo patterning. Development.

[18] Costantini F, Kopan R (2010). Patterning a complex organ: branching morphogenesis and nephron segmentation in kidney development. Dev. Cell.

[19] Cederquist GY (2019). Specification of positional identity in forebrain organoids. Nat. Biotechnol..

[20] Ben-Reuven L, Reiner O (2020). Toward spatial identities in human brain organoids-on-chip induced by morphogen-soaked beads. Bioengineering.

[21] Demers CJ (2016). Development-on-chip: in vitro neural tube patterning with a microfluidic device. Development.

[22] Amadi OC (2010). A low resistance microfluidic system for the creation of stable concentration gradients in a defined 3D microenvironment. Biomed. Microdevices.

[23] Park JY (2009). Differentiation of neural progenitor cells in a microfluidic chip-generated cytokine gradient. Stem Cells.

[24] Wang Y (2017). A microengineered collagen scaffold for generating a polarized crypt-villus architecture of human small intestinal epithelium. Biomaterials.

[25] Manfrin A (2019). Engineered signaling centers for the spatially controlled patterning of human pluripotent stem cells. Nat. Methods.

[26] Ahmad AA, Wang Y, Sims CE, Magness ST, Allbritton NL (2015). Optimizing Wnt-3a and R-spondin1 concentrations for stem cell renewal and differentiation in intestinal organoids using a gradient-forming microdevice. RSC Adv..

[27] Hagiwara M, Nobata R, Kawahara T (2018). High repeatability from 3D experimental platform for quantitative analysis of cellular branch pattern formations. Integr. Biol..

[28] Hagiwara M, Kawahara T, Nobata R (2016). Tissue in cube: in vitro 3D culturing platform with hybrid gel cubes for multidirectional observations. Adv. Health. Mater..

[29] Rogers KW, Schier AF (2011). Morphogen gradients: from generation to interpretation. Annu. Rev. Cell Dev. Biol..

[30] Hofer M, Lutolf MP (2021). Engineering organoids. Nat. Rev. Mater..

[31] Alves-Lopes JP, Söder O, Stukenborg JB (2017). Testicular organoid generation by a novel in vitro three-layer gradient system. Biomaterials.

[32] Vetter R, Iber D (2022). Precision of morphogen gradients in neural tube development. Nat. Commun..

[33] Matos, I. et al. Progenitors oppositely polarize WNT activators and inhibitors to orchestrate tissue development robin chemers neustein laboratory of mammalian cell biology and development. *Elife* **9**, e54304 (2020). 10.7554/eLife.54304 PMC7224699 32310087

[34] Carlson, B. *Human Embryology and Developmental Biology* (Elsevier, 2018).

[35] Lam AQ (2014). Rapid and efficient differentiation of human pluripotent stem cells into intermediate mesoderm that forms tubules expressing kidney proximal tubular markers. J. Am. Soc. Nephrol..

[36] Bianchi F (2018). Rapid and efficient differentiation of functional motor neurons from human iPSC for neural injury modelling. Stem Cell Res..

[37] Stapornwongkul KS, Vincent JP (2021). Generation of extracellular morphogen gradients: the case for diffusion. Nat. Rev. Genet..

[38] Amsden B (1998). Solute diffusion within hydrogels. Mechanisms and models. Macromolecules.

[39] Müller P (2012). Differential diffusivity of nodal and lefty underlies a reaction-diffusion patterning system. Science.

[40] Kicheva A (2007). Kinetics of morphogen gradient formation. Science.

[41] Yu SR (2009). Fgf8 morphogen gradient forms by a source-sink mechanism with freely diffusing molecules. Nature.

[42] Burla F, Sentjabrskaja T, Pletikapic G, van Beugen J, Koenderink GH (2020). Particle diffusion in extracellular hydrogels. Soft Matter.

[43] Lieleg O, Baumgärtel RM, Bausch AR (2009). Selective filtering of particles by the extracellular matrix: an electrostatic bandpass. Biophys. J..

[44] Axpe E (2019). A multiscale model for solute diffusion in hydrogels. Macromolecules.

[45] Ibañes M, Belmonte JCI (2008). Theoretical and experimental approaches to understand morphogen gradients. Mol. Syst. Biol..

[46] Green JBA, Sharpe J (2015). Positional information and reaction-diffusion: two big ideas in developmental biology combine. Development.

[47] Spurlin JW, Nelson CM (2017). Building branched tissue structures: from single cell guidance to coordinated construction. Philos. Trans. R. Soc. B Biol. Sci..

[48] Shao Y, Fu J (2022). Engineering multiscale structural orders for high-fidelity embryoids and organoids. Cell Stem Cell.

[49] Hagiwara M, Nakase I (2018). Epidermal growth factor induced macropinocytosis directs branch formation of lung epithelial cells. Biochem. Biophys. Res. Commun..

[50] Chumbimuni-Torres KY (2011). Adsorption of proteins to thin-films of PDMS and its effect on the adhesion of human endothelial cells. RSC Adv..

[51] Toepke MW, Beebe DJ (2006). PDMS absorption of small molecules and consequences in microfluidic applications. Lab Chip.

[52] Nianzhen LI (2009). PDMS compound adsorption in context. J. Biomol. Screen..

[53] Gökaltun A (2019). Simple surface modification of poly(dimethylsiloxane) via surface segregating smart polymers for biomicrofluidics. Sci. Rep..

[54] Ren K, Zhao Y, Su J, Ryan D, Wu H (2010). Convenient method for modifying poly(dimethylsiloxane) to be airtight and resistive against absorption of small molecules. Anal. Chem..

[55] Ishihara K, Fukumoto K, Iwasaki Y, Nakabayashi N (1999). Modification of polysulfone with phospholipid polymer for improvement of the blood compatibility. Part 1. Surface characterization. Biomaterials.

[56] Shin S, Kim N, Hong JW (2018). Comparison of surface modification techniques on polydimethylsiloxane to prevent protein adsorption. Biochip J..

[57] Blin G (2018). Geometrical confinement controls the asymmetric patterning of brachyury in cultures of pluripotent cells. Development.

[58] Gjorevski N (2022). Tissue geometry drives deterministic organoid patterning. Science.

[59] Takano A, Koh I, Hagiwara M (2022). 3D culture platform for enabling large-scale imaging and control of cell distribution into complex shapes by combining 3D printing with a cube device. Micromachines.

[60] Nielsen JB (2020). Microfluidics: innovations in materials and their fabrication and functionalization. Anal. Chem..

[61] Ren K, Zhou J, Wu H (2013). Materials for microfluidic chip fabrication. Acc. Chem. Res..

