# Supplementary material for: Gradient to sectioning CUBE workflow for the generation and imaging of organoids with localized differentiation
Source: Commun Biol. 2023 Mar 21;6:299. doi: 10.1038/s42003-023-04694-5 (PMC10030548; doi:10.1038/s42003-023-04694-5)
Supplement: Supplementary file 1 — Supplementary Figures [file 42003_2023_4694_MOESM1_ESM.pdf]

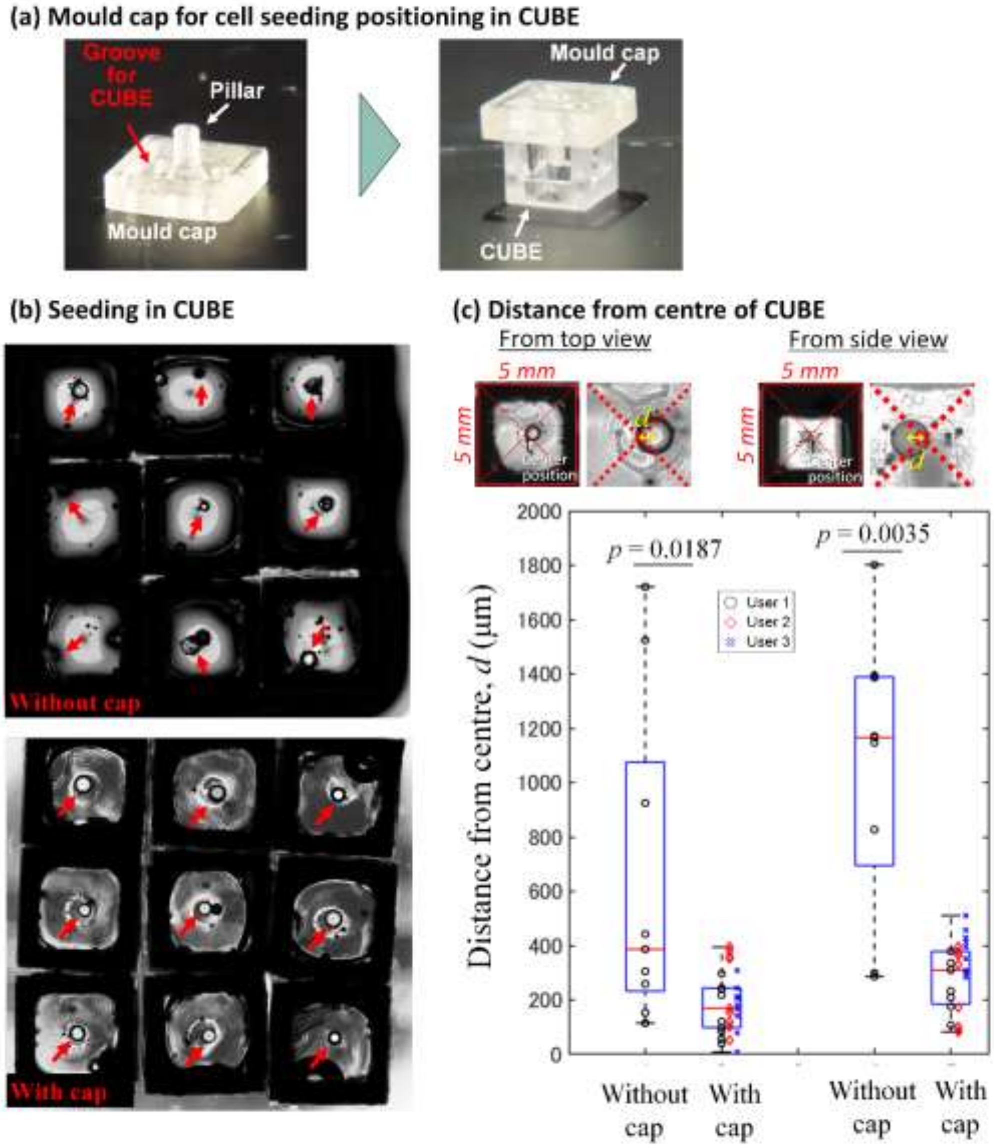

Supplementary Figure 1. Controlling the seeding position of cells in the CUBE. (a) By placing a mould cap in the hydrogel in the CUBE, a seeding pocket is formed in the gel after it has cured and the mould cap is removed. (b) Top view of brightfield images of PDMS spheres seeded in Cryo CUBEs with and without the use of the mould cap to represent hiPSC spheroid seeding. Red arrows indicate the position of the PDMS spheres in the CUBE. (c) Measurements of the distance of PDMS sphere from the centre of the CUBE, from both the top view and side view of the CUBE, show that seeding in the pocket created by the mould cap resulted in consistent seeding position close to the centre of the CUBE, compared to manual seeding without a guide. To demonstrate the ease of controlling the seeding position by using the mould cap method, the experiment was performed by User 1 who is experienced in seeding spheroids in CUBEs, and by Users 2 (volunteer junior high student) and 3 (new postdoc) who have no experience in seeding spheroids. The results show that, with minimal training, even inexperienced users can easily seed spheroids at the desired position in the CUBE significantly more accurately and precisely than an experienced user without the mould cap. The elements in the boxplots are defined as follows: centre line; median; box limits, upper and lower quartiles; whiskers, 1.5x interquartile range. p value was calculated by Kolmogorov-Smirnov (KS) test; n=9 for each user.

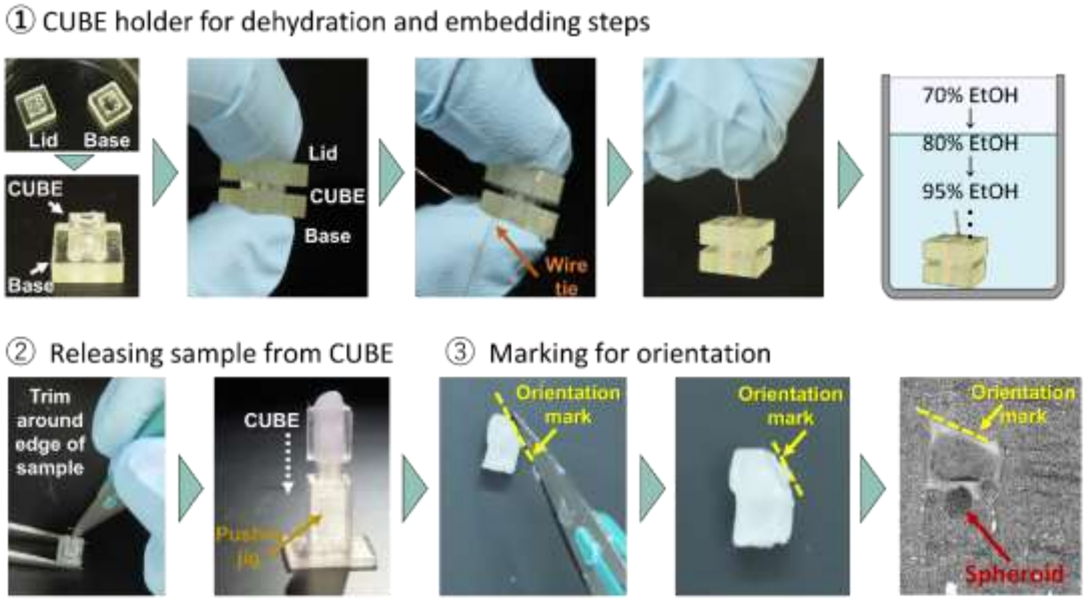

Supplementary Figure 2. Detailed process showing the preparation of sample for paraffin sectioning. A CUBE holder was designed to prevent the risk of losing samples during the paraffinization process. After paraffinization, the sample is released from the CUBE and marked for orientation by cutting one edge of the sample.

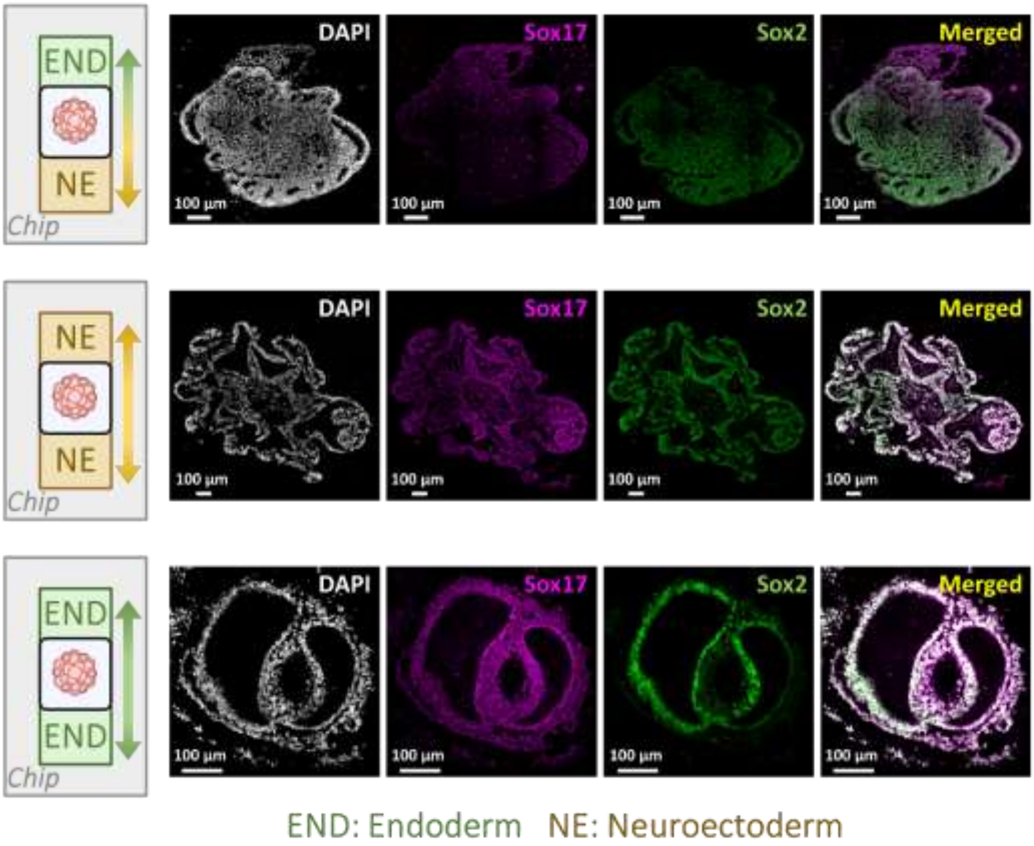

Supplementary Figure 3. Immunofluorescence of paraffin-sectioned sample of END-NE differentiated spheroid. Immunostaining showed localized expression pattern of endoderm (Sox17) and neuroectoderm (Sox2) markers in END-NE spheroids, whereas NE only and END only controls showed uniform distribution of the markers.

Supplementary Table 1. List of antibodies for immunofluorescence staining

Reagents	Source and Identifier	Dilution
Mouse monoclonal Anti-Sox2	Abcam, ab79351	1:200 in IF+G
Rabbit monoclonal Anti-Nestin	Abcam, ab105389	1:200 in IF+G
Goat polyclonal Anti-Brachyury	R&D Systems, AF2085	1 μg/mL in IF+0.1% BSA
Goat polyclonal Anti-FoxA2	R&D Systems, AF2400	1 μg/mL in IF+0.1% BSA
Goat polyclonal Anti-Sox17	R&D Systems, AF1924	1 μg/mL in IF+0.1% BSA
Donkey anti-goat AF555	Invitrogen, A32816	1:200 in IF+0.1% BSA
Goat anti-mouse AF488	Invitrogen, A32723	1:200 in IF+G
Goat anti-rabbit AF488	Invitrogen, A32731	1:200 in IF+G
DAPI	Invitrogen, D1306	600 nM in DPBS
