# Supplementary material for: Gradient to sectioning CUBE workflow for the generation and imaging of organoids with localized differentiation
Source: Commun Biol. 2023 Mar 21;6:299. doi: 10.1038/s42003-023-04694-5 (PMC10030548; doi:10.1038/s42003-023-04694-5)
Supplement: Supplementary file 2 — Description of Additional Supplementary Files [file 42003_2023_4694_MOESM2_ESM.pdf]

Description of Additional Supplementary Files

File name: Supplementary Data 1
Description: Source data underlying Figures 3c and Supplementary Figure 1c.

File name: Supplementary Movie 1
Description: Experimental procedure to show how the CUBE is integrated with the chip.
